# Group B streptococcal empyema necessitatis with pleural fistula after blunt trauma: A case report

**DOI:** 10.1016/j.ijscr.2019.09.006

**Published:** 2019-09-18

**Authors:** Takashi Ohki, Yoshiki Shigematsu, Shunzo Hatooka

**Affiliations:** Department of Respiratory Surgery, Ichinomiya-Nishi Hospital, 1 Kaimei Hira, Ichinomiya-shi, Aichi, Japan

**Keywords:** CT, computed tomography, EN, empyema necessitatis, GBS, group B *Streptococcus*, HbA1c, glycated hemoglobin, Empyema necessitatis, Septic arthritis, Group B *Streptococcus*, Blunt trauma

## Abstract

**Introduction:**

We report the first case of empyema necessitatis (EN) with pleural fistula and septic arthritis caused by *Streptococcus agalactiae* following blunt trauma.

**Presentation of the case:**

A 46-year-old man with diabetes mellitus and a history of recent right rib fracture and right knee bruising presented with dyspnea and right knee pain. He was diagnosed with EN and underwent chest drainage, followed by open-window thoracotomy. Septic arthritis occurred on day 8 after thoracotomy. The chest wall wound healed after 3 months.

**Discussion:**

EN is a rare complication of empyema. In this patient, infection was invasive, causing necrotizing pneumonia with a pleural fistula. To our knowledge, there are no reports of group B streptococcal EN with a pleural fistula resulting from blunt chest trauma.

**Conclusion:**

Group B streptococcal infection might become invasive in immunocompromised patients, so careful follow-up for those patients is important.

## Highlights

• Empyema necessitatis (EN) occurred with a pleural fistula after blunt chest trauma.
• We report the first case of group B streptococcal (GBS) EN resulting from trauma.
• Septic arthritis occurred after open-window thoracotomy.
• GBS infection might become invasive in immunocompromised patients.
• Careful follow-up of such patients who develop a new focus of infection is important.

## 1 Introduction

Empyema necessitatis (EN) is a rare complication of empyema in which the pleural infection spreads outside the pleural space to involve the soft tissues of the chest wall. It is often caused by *Mycobacterium tuberculosis* infection [1]. *Streptococcus agalactiae* (group B *Streptococcus*; GBS) is a very rare entity causing EN, with only two cases reported to date [2,3]. Here, we report a case of EN with pleural fistula and septic arthritis of the knee following a rib fracture and knee bruising following blunt trauma. This case report has been written in line with the SCARE guidelines [4].

## 2 Presentation of the case

**Fig. 1:**
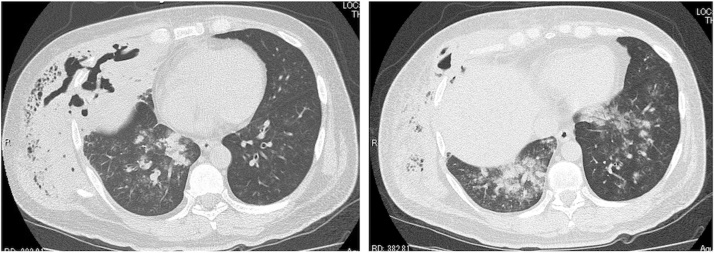
Chest computed tomography (CT) on day 1.

A 46-year-old man was admitted to the emergency department of our hospital because of dyspnea and right knee pain. He had fallen and bruised his right chest 2 weeks before. He was diagnosed with a fracture to his right sixth rib at an orthopedic clinic, and a chest band and analgesics were prescribed. A week later, he fell down again and bruised his right knee. No fracture of the right knee was noted. He had a history of diabetes but had discontinued treatment for 6 months. His height was 167.3 cm, weight 73.4 kg, and body mass index (BMI) 26.2 kg/m^2^. His blood pressure was 147/73 mmHg, pulse 142 beats per minute, temperature 39.9 °C, and oxygen saturation 85% while he was breathing ambient air. There were swelling and pain on the right side of his chest. Slight swelling was recognized on the right knee, although joint fluid culture was negative. The patient had no history of smoking. Laboratory data showed a white cell count of 13,800/mm^3^, a C-reactive protein level of 296 mg/l, a serum glucose level of 462 mg/dl, and a glycated hemoglobin (HbA1c) concentration of 9.9%. Chest computed tomography (CT) scans on admission are shown in Fig. 1. These showed the fracture of the right sixth rib, a partially destroyed right middle lobe of the lung, and an extensive abscess through the right chest wall. Consolidation and ground-glass opacities in both lower lobes of the lungs suggestive of aspiration pneumonia were also noted.

**Fig. 2:**
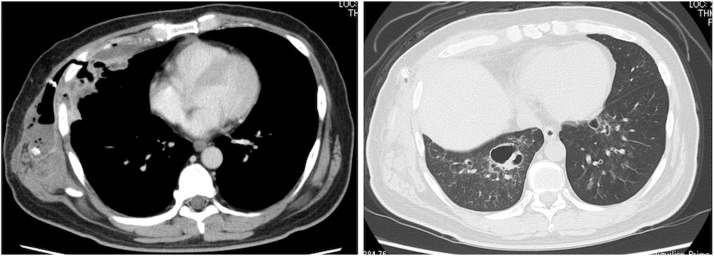
Chest computed tomography on day 11 before open-window thoracotomy.

The patient was diagnosed with EN, necrotizing pneumonia, and a right sixth rib fracture and was admitted to the respiratory department (day 1). Treatment with intravenous antibiotics with ampicillin plus sulbactam and clindamycin was started. A chest tube was inserted and the abscess fluid was submitted to the laboratory for bacterial culture. An acid-fast bacillus smear test was negative. Continuous negative pressure drainage with a chest tube was started. This showed a small amount of air leakage but it became minimal from day 2. His sputum was submitted for bacterial culture. His blood glucose was strictly controlled with insulin. The patient’s fever subsided immediately after treatment was started, but went up over 38 °C again on day 4. Because GBS could be grown from both sputum and abscess fluids, ampicillin with sulbactam was de-escalated to ampicillin from day 7. The fever reduced temporarily, but rose again on day 11, and a second chest CT was performed (Fig. 2). An abscess on the right chest wall and empyema remained ventral to the rib fracture. The pneumonia in both lungs improved. He was referred to our department on day 12 for surgical treatment. An open-window thoracotomy was planned on day 13.

**Fig. 3:**
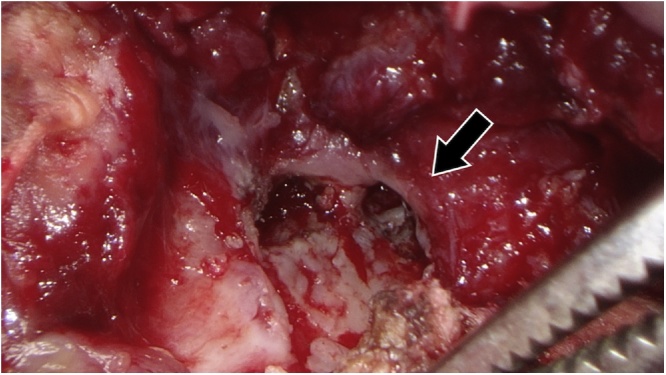
A pleural fistula was found during surgery (arrow).

**Fig. 4:**
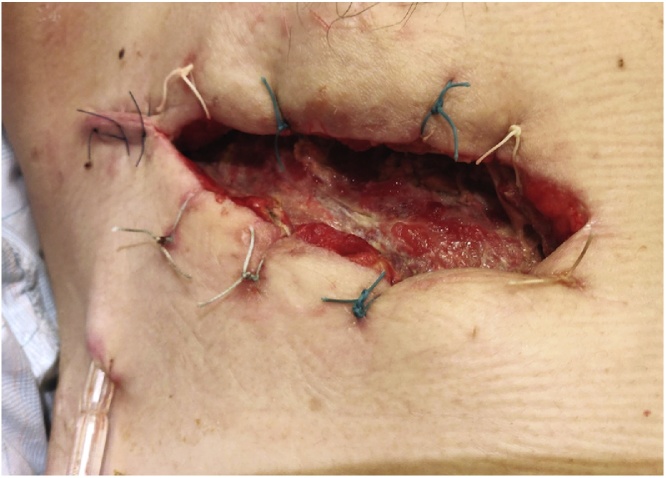
Appearance of wound after open-window thoracotomy.

Surgery was performed with the patient in the left half-lateral decubitus position under general anesthesia. A 13-cm incision was made on the site of the rib fracture. The sixth rib was resected 7 cm around the fracture because it had formed a sequestrum. An abscess below the rib was opened. A pleural fistula was found just below the resected rib and air leakage was observed (Fig. 3). The abscess on the ventral side of the chest wall was also opened. The subfascial abscess cavity was also cleaned and closed (Fig. 4). The operation time was 110 min and the blood loss was 150 ml.

**Fig. 5:**
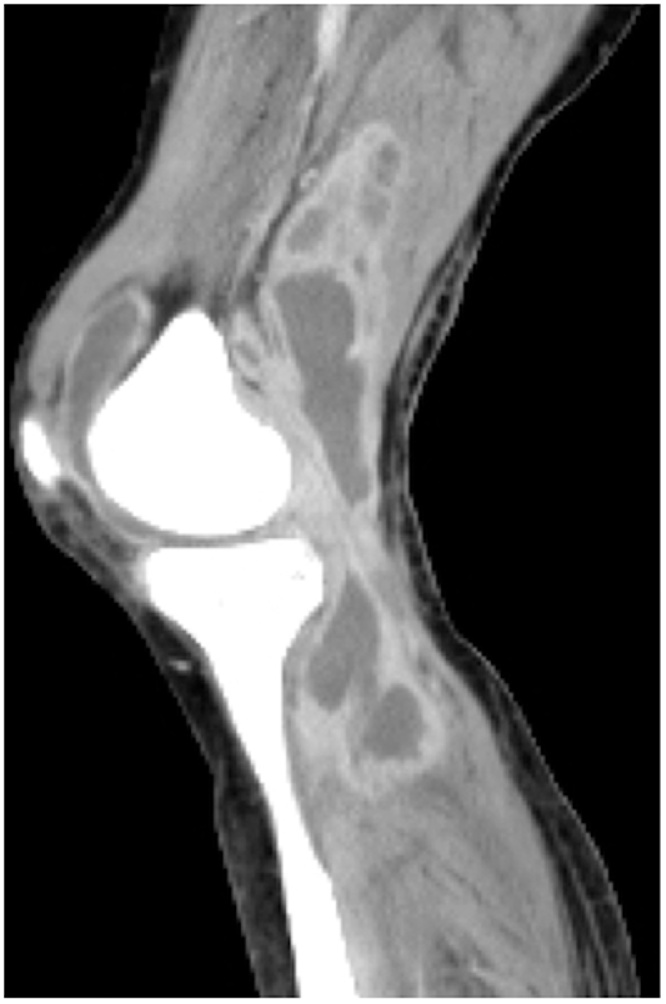
Contrast-enhanced CT of the patient’s right knee on day 22.

Changing the packing gauze and continuous negative pressure drainage of the subfascial cavity were continued. Administration of ampicillin was also continued. Both the fever and inflammatory responses tended to improve, but on day 21 fever returned. Another infection outside the surgical site was suspected, and CT from the neck to the lower limbs was performed on day 22 (Fig. 5). An abscess around the right knee joint was noted and septic arthritis was suspected. We referred the patient to the orthopedic department, and he underwent surgical debridement and arthroscopic synovectomy of the right knee joint. The wound was closed and a drain was placed. Sulfamethoxazole, trimethoprim, and rifampicin were prescribed additionally. Bacterial cultures of the right knee abscess fluids were negative. Thereafter, the fever and laboratory data improved, and the patient was discharged on day 36. Three months after the thoracotomy, the wound was fully epithelialized.

## 3 Discussion

GBS is known to cause a variety of invasive infections in adults [[5], [6], [7]], and obese or patients with diabetes have an increased risk of invasive infection [8]. To our knowledge, there are no reports of GBS leading to EN resulting from blunt chest trauma. The keywords “(empyema necessitatis) OR (empyema necessitans)” were used to search the PubMed database for relevant articles published before 2019. It is worth noting that this patient with poorly controlled diabetes had complicated necrotizing pneumonia with a pleural fistula, aspiration pneumonia, and septic arthritis.

A pleural fistula was found during surgery, but air leakage was minimal before the surgery. This was probably because the drainage was ineffective due to sticky pus. Cases of empyema with a pleural fistula are usually treated with an endoscopic approach or surgical treatment including closure of a fistula with a vascularized flap [9], while it can be treated successfully using conservative management [10]. Negative pressure wound therapy could shorten the time to close the open-window thoracotomy wound [11], but our patient did not accept it for personal reasons.

The chest wall infection had been controlled successfully and we were able to diagnose septic arthritis immediately when the patient’s fever went up after open-window thoracotomy. Septic arthritis occurs at a frequency of 2–10/10 million, and various joints are involved. Symptoms and signs of this disorder are an important medical emergency, with high morbidity and mortality rates [12]. *Staphylococcus aureus* is the most common cause of septic arthritis, whereas GBS is a relatively uncommon entity. Older age, high C-reactive protein level, and diabetes mellitus are risk factors for higher mortality [13]. Septic arthritis in this patient was possibly caused by GBS, but the actual cause is unknown because culture of fluid drained from the knee abscess was negative.

## 4 Conclusion

We performed an open-window thoracotomy for EN with pleural fistula after a blunt chest trauma. GBS is a very rare causative entity for this condition, but the infection might become invasive in immunocompromised patients. Careful follow-up of those high-risk patients who might have a new focus of infection is important.

## Sources of funding

This research did not receive any specific grant from funding agencies in the public, commercial, or not-for-profit sectors.

## Ethical approval

The IRB committee of our hospital decided that approval is not necessary for this case.

## Consent

Written informed consent was obtained from the patient for the publication of this case report and any accompanying images.

## Author contribution

Takashi Ohki: Conceptualization, writing original draft, review, and editing.

Yoshiki Shigematsu: Writing, review, and editing.

Shunzo Hatooka: Writing, review, and editing.

## Registration of research studies

Not applicable.

## Guarantor

Shunzo Hatooka.

## Provenance and peer review

Not commissioned, externally peer-reviewed.

## Declaration of Competing Interest

The authors have no conflicts of interest to declare.
